# Deep RetinaNet-Based Detection and Classification of Road Markings by Visible Light Camera Sensors

**DOI:** 10.3390/s19020281

**Published:** 2019-01-11

**Authors:** Toan Minh Hoang, Phong Ha Nguyen, Noi Quang Truong, Young Won Lee, Kang Ryoung Park

**Affiliations:** Division of Electronics and Electrical Engineering, Dongguk University, 30 Pildong-ro 1-gil, Jung-gu, Seoul 100-715, Korea; hoangminhtoan@dongguk.edu (T.M.H.); phongnhhn92@gmail.com (P.H.N.); noitq.hust@gmail.com (N.Q.T.); lyw941021@dongguk.edu (Y.W.L.)

**Keywords:** detection and classification of road markings, deep CNN, one-stage RetinaNet, NVIDIA Jetson TX2, visible light camera sensor

## Abstract

Detection and classification of road markings are a prerequisite for operating autonomous vehicles. Although most studies have focused on the detection of road lane markings, the detection and classification of other road markings, such as arrows and bike markings, have not received much attention. Therefore, we propose a detection and classification method for various types of arrow markings and bike markings on the road in various complex environments using a one-stage deep convolutional neural network (CNN), called RetinaNet. We tested the proposed method in complex road scenarios with three open datasets captured by visible light camera sensors, namely the Malaga urban dataset, the Cambridge dataset, and the Daimler dataset on both a desktop computer and an NVIDIA Jetson TX2 embedded system. Experimental results obtained using the three open databases showed that the proposed RetinaNet-based method outperformed other methods for detection and classification of road markings in terms of both accuracy and processing time.

## 1. Introduction

**Figure 1:**
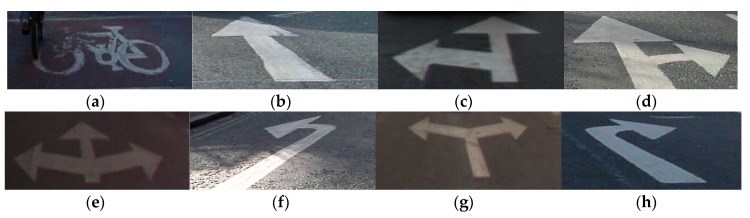
Road marking objects. (**a**) Bike. (**b**) Forward arrow. (**c**) Forward-left arrow. (**d**) Forward-right arrow. (**e**) Forward-left-right arrow. (**f**) Left arrow. (**g**) Left-right arrow. (**h**) Right arrow.

**Figure 2:**
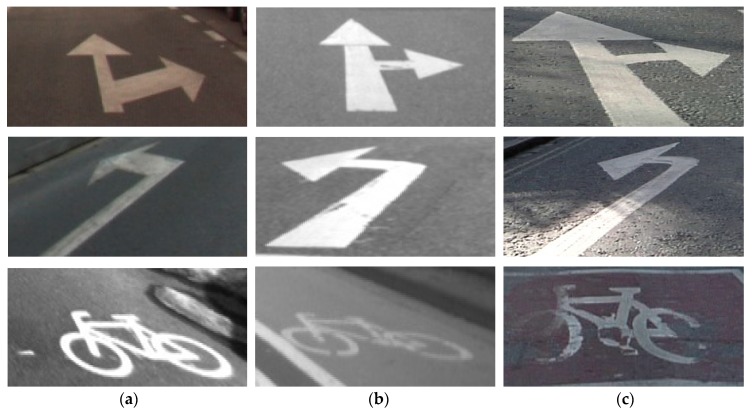
Different shapes of arrow markings observed in the image from the front-view camera in the vehicle for different datasets. (**a**) Malaga urban dataset image captured in Spain [16]. (**b**) Daimler dataset image captured in Germany [17]. (**c**) Cambridge dataset image captured in UK [18].

The U.S House of Representatives is quoted to have said “Self-driving cars seem like such a good idea that even Republicans and Democrats can agree on their merits” [1]. Autonomous vehicles are considered as the future of mobility. The most essential requirement for robust advanced driver assistance systems (ADAS) is to make the perception of the environment around the vehicle as comprehensive as possible. Although road lane markings can be defined by a combination of horizontal and vertical lines, arrow markings vary. Arrow markings have different signature features such as straight forward, left, right, forward-left-right arrow, or different color intensities even within the same city or different character sets depending on the countries. The sizes of arrow markings also vary when considering the distance and angular orientation of the front-view camera in the vehicle. Therefore, the same arrow can show different lengths or thickness in different frames. Furthermore, recognition of arrow markings becomes increasingly difficult because of occlusion. For example, forward-left or forward-right arrow can be easily mischaracterized as forward arrow in case the left or right part, respectively, is faint owing to factors such as shadows from nearby cars and trees or paint quality. Figure 1 shows examples of road markings of a bike, forward arrow, forward-left arrow, forward-right arrow, forward-left-right arrow, left arrow, left-right arrow, and right arrow. Figure 2 shows examples of different shapes of arrow markings observed in the image obtained using a front-view camera in a vehicle on different datasets.

Conventional methods that do not employ machine learning techniques [2,3,4,5] require additional pre- and post-processing steps to transform the input image for increasing the contrast between the road markings and the background [6]. However, these methods only tackle problems such as illumination variations [7,8], curve line, or color intensities. These methods are not generalized for all application to all challenges. During the past few decades, deep learning has shown significant performance owing to the capabilities of parallel computing using graphics processing units (GPUs) and a breakthrough of huge collected and labeled data [9,10]. Based on these advancements, methods [11,12,13,14] based on a deep learning have been developed to tackle the above-mentioned challenges in road markings recognition. These methods have demonstrated a high performance on benchmarks and in real road scenarios. Applying the advantages of visual road understanding based on deep learning, we propose the detection and classification of road markings using a one-stage convolutional neural network (CNN), called RetinaNet [15] that works well in various complex environmental conditions as well as for small markings at far distance.

The remainder of this paper is organized as follows: Section 2 describes related works on detection and classification of road markings. Our contributions are listed in Section 3. In Section 4, the proposed method is explained in detail. Section 5 presents the experimental setup and the results. Our conclusions and discussions on ideas for future work are reported in Section 6**.**

## 2. Related Works

Previous studies on detection and classification of road markings are categorized into handcrafted features-based and deep features-based methods. In the former category of methods, Li et al. [6] combined a local adaptive threshold and canny edge detection for extraction of road markings. However, the performance of their method relied on the accuracy of the canny edge detector. Yoo et al. [7] converted the color space from red-green-blue (RGB) to luminance-chroma blue-chroma red (YCbCr) for gradient-enhancing to deal with illumination changes. They assumed that multiple different illuminations were not included within a single scene; thus, their method was only effective for limited variations of illumination. Instead of using YCbCr color space, Sun et al. [8] chose the HSI color representation with a Fuzzy c-means algorithm, and simple thresholds were empirically selected for saturation and intensity values to detect lane markings. In [2,3], the authors used method based on a line segment detector, which can be operated regardless of the orientation or size of line segment. However, their methods require a distinct contrast to exist between the road lane marking and the background for accurate edge detection. 

The performances of the abovementioned handcrafted features-based methods are limited in complex road environments. Therefore, deep features-based methods have been studied. Chen et al. [19] proposed a general framework for road marking detection and classification by using binarized normed gradient (BING) and principal component analysis network (PCANet). The BING object detector provides a number of possible candidate regions that have relevant similarities to road markings, the detected regions are then classified by PCANet. However, the drawback of their approach is that the number of candidate regions determined by the BING detector causes a computational burden for the classification process using PCANet. In addition, road markings are not localized precisely; hence, the bounding box often includes other irrelevant objects. Lee et al. [14] used the vanishing point guided net (VPGNet) model for lane and road markings detection and recognition under adverse weather conditions. They evaluated their network model using images captured from a downtown area of Seoul, South Korea. These images had a resolution of 1288 × 728 pixels. Thus, all the shapes and symbols of the lane and road markings in the images follow the regulations of South Korea. Although they are different from those in other open datasets captured from different countries, they did not evaluate their method with these datasets. Li et al. [12] used a CNN and a recurrent neural network (RNN) to detect the boundaries of road lane marking. In their study, the multi-task CNN provides geometric information of the given lane structures, and the RNN automatically detects lane boundaries without any explicit prior knowledge or secondary modeling. He et al. [20] proposed a method using a dual-view CNN (DVCNN) framework for the detection of road lane marking. In their approach, both the images of front-view and top-view were fed as inputs to the DVCNN. Distractions from moving vehicles, barriers, and curbs are excluded from the front-view image, and the club-shaped structures were maintained in the top-view image. Unfortunately, their method shows low accuracy in case that the road lane marking is occluded by the other vehicles or the image is completely over-exposed. Huval et al. [21] presented empirical evaluations of the detection of road lane marking and vehicle marking based on computer vision algorithms combined with deep learning. Their network includes sub-networks that perform binary classification and regression tasks. However, the results were evaluated on highway images without complex road markings or occlusion from other vehicles. Al-Qizwini et al. [22] proposed a deep learning-based algorithm for autonomous vehicles using GoogLeNet for autonomous driving (GLAD). However, their algorithm was evaluated using images generated by the open racing car simulator (TORCS) instead of camera images from a real vehicle environment. For the detection and classification of road markings, Bailo et al. [23] presented a technique using density-based grouping based on maximally stable extremal regions (MSER) features to obtain candidate regions. Then, the regions of interest (ROIs) were recognized using a shallow CNN comprising a single convolutional layer, 1 max pooling layer, and 3 fully connected layers. However, to recognize the road marking object using their CNN model, their algorithm relies on an MSER detector to detect the correct ROI candidates by pre-processing, including image rectification and enhancement, to increase the distinctiveness between objects and their background. Thus, the enhanced images of the road might include excessive noisy texture information, which could reduce the accuracy in the classification of road markings. To overcome the limitations of previous studies, we propose the detection and classification method of various types of road markings on roads in various complex environments using a one-stage deep CNN, called RetinaNet. In Table 1, we summarize the comparisons between the proposed method and existing methods.

## 3. Contributions

Below, we summarize the novelty of this study in five points.

- This is the first approach using one-stage deep CNN for the detection and classification of road markings. This method achieved high accuracies of detection and classification in complex conditions such as extreme illumination change, occlusion, and far distance.
- The proposed system does not require any pre-processing including image rectification and enhancement, or post-processing for the detection and classification of road markings.
- We determined that a converted bird’s eye view image cannot cover all drivable regions where some part of original road markings disappear. This negatively influences the training of the CNN model.
- Considering the application of autonomous vehicles in real environments, we tested the trained CNN model not only on a desktop computer but also on an NVIDIA Jetson TX2 embedded system [24], which has been widely used as onboard platform in autonomous vehicles.
- Finally, although the open databases used in our experiments have been widely used in previous studies, they do not provide annotated information of road markings. This increases the time and load for system implementation. Therefore, we provide the manually annotated information of road markings for the Malaga urban dataset, the Daimler dataset, and the Cambridge dataset as shown on the website [25]. We also provide the proposed train models based on different backbones with and without pre-trained weights to other researchers for fair comparison.

## 4. Proposed Method Using Deep RetinaNet

### 4.1. Overview of Proposed Method

**Figure 3:**
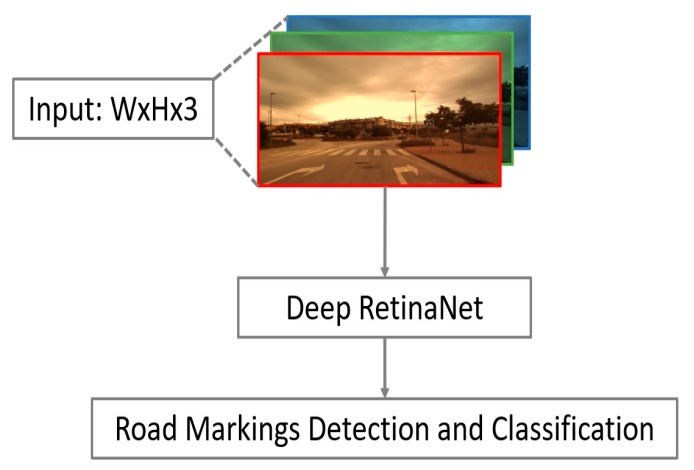
Proposed method for the detection and classification of road markings based on deep RetinaNet.

Figure 3 shows the overall flowchart of proposed method. The input image from three channels is captured using a front-view camera mounted on the car, and it is used as an input for deep RetinaNet. From the outputs of RetinaNet, the positions and classes of road markings are determined. As shown in Figure 3, our method does not require pre- and post-processing.

### 4.2. Architecture of the Deep RetinaNet Model

**Figure 4:**
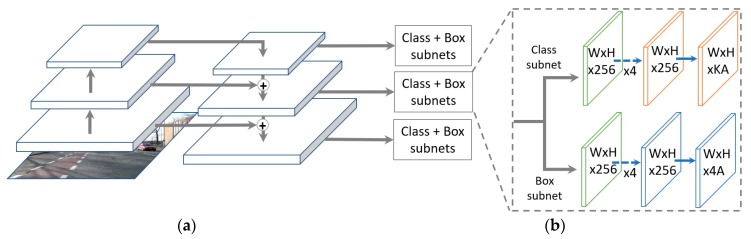
Architecture of RetinaNet: (**a**) to produce the multi-scale convolutional feature pyramid using a residual network (ResNet) as an encoder (left) and a feature pyramid net (FPN) as a decoder (right). (**b**) Class subnet for classifying anchor boxes (top), and box subnet for regressing from anchors boxes to ground-truth object boxes (bottom).

The architectures for networks tasked with object detection is usually split in two categories, namely single-stage (or one-stage) and two-stage object detectors [26]. In two-stage detectors such as R-CNN [27], Faster region-based CNN (R-CNN) [28] and Mask R-CNN [29], a region proposal network is used (RPN) to generate ROIs in the first stage. Subsequently, these ROI proposals are transferred down the pipeline for object classification and bounding-box regression in the second stage. These two-stage models are very slow; however, they yield a high accuracy because they maintain a manageable balance between the foreground and the background. On the other hand, one-stage detectors such as you only look once (YOLO)v3 and single shot multibox detector (SSD) [30,31] do not have a pre-selection step for detection of foreground candidates and they treat object detection as a simple regression problem. These one-stage methods normally use 10,000~100,000 box proposals per image, compared to only 2000 proposals generated by two-stage methods like Faster R-CNN [32]. Therefore, they yield a lower detection accuracy; however, they are faster than two-stage object detectors. Our system for detecting and classification for road markings is built to be operated on an embedded system in an actual car, which usually has lower computing power than a desktop computer. By considering the aspects of processing speed and accuracy, we use the one-stage object detection based on deep RetinaNet architecture as shown in Figure 4 [15].

**Figure 5:**
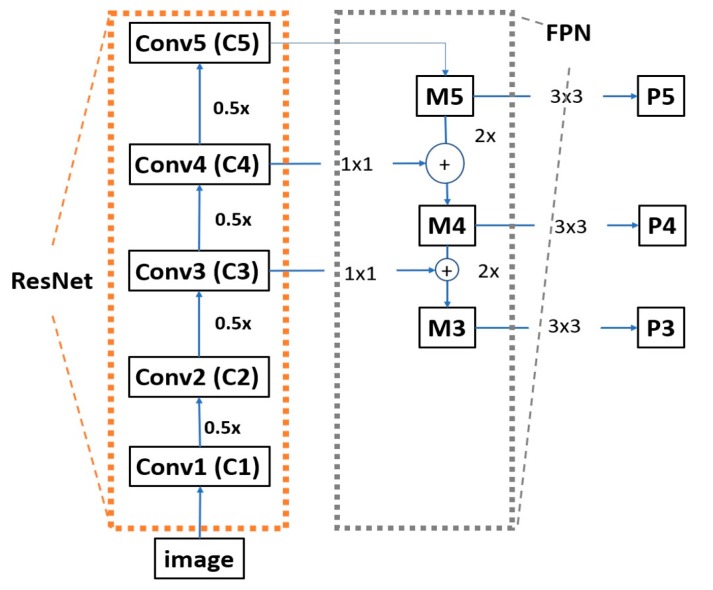
Architecture of deep RetinaNet with revised ResNet (block in orange box) and FPN (block in gray box). M3~5 means the feature maps obtained from Conv3~5, respectively, whereas P3~5 shows the feature maps for prediction.

The backbone is necessary for computing a convolutional feature map over the entire input image. It consists of an encoder and a feature pyramid net (FPN) including subnets [33]. The original road scene image can be applied as input to a residual network (ResNet) [34], dense convolutional network (DenseNet) [35], or visual geometry group (VGG) net [36] encoder, which processes the image through convolution kernels and generates deep features. Each component of the ResNet architecture [34], which is the backbone of deep RetinaNet, is explained in detail as follows. The ResNet model enables training hundreds of layers while still maintaining compelling performance, and the performance of many computer vision applications and image classification schemes have been improved. In our research, we can process a 3-channel image regardless of its size, and we only need to specify the number of channels as an input parameter. The size of the output feature map can be calculated using Equations (1) and (2) below [37,38]:

(1) $$output\;height=\frac{H-F_{h}+2P}{S_{h}}+1$$

(2) $$output\;width=\frac{H-F_{w}+2P}{S_{w}}+1$$

where H, W, $F_{h}$, $F_{w}$, $S_{h}$, and $S_{w}$ are the dimensions of the input image (height = H, width = W), a filter (height = $F_{h}$, width = $F_{w}$), and stride (height = $S_{h}$, width = $S_{w}$), respectively. *P* is the number of padding. The bottom-up pathway uses ResNet50 as the encoder, as shown in the left structure of Figure 4a and is composed of many convolution modules; each module has several convolutional layers. As shown in Table 2, ResNet50 without the last average pooling layer, fully connected layer, and softmax layer is used in our RetinaNet. As we move up from lower to higher modules in ResNet50 of Figure 4a, the spatial dimensions are reduced by half. The output of each last residual block is labeled as Ci (i varies from 1 to 5), and both C1 and C2 are not connected to the FPN because of its large memory footprint [33], as shown in Figure 5.

Instead of adding a classifier right after ResNet50, FPN is used as a decoder [33]. The advantages of using FPNs are that feature maps can be chosen from different layers of ResNet50; therefore, rich and multi-scaled features can be obtained. Because objects appear in various scales and sizes, an image pyramid is used to make it easy for CNN-based object detection. Therefore, some of the reported studies used only a single scale prediction, whereas others obtained predictions from intermediate layers.

**Figure 6:**
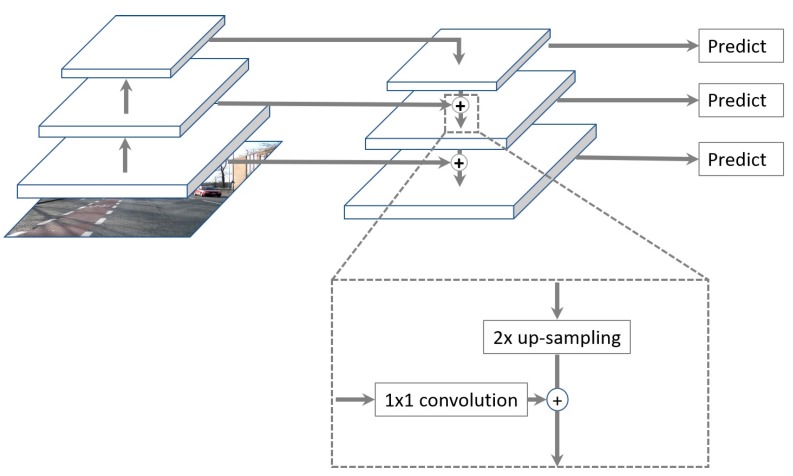
Lateral connections between the ResNet backbone and FPN, and top-down pathway merged by addition.

Unlike these approaches, an FPN uses simple merge layers (mode = “addition”) to combine both, as illustrated in Figure 5 and Figure 6. For each feature map, the FPN up-samples the spatial resolution of the input feature map by a factor of two, and the up-sampled map is then merged with the corresponding bottom-up map, which undergoes a 1 × 1 convolution to reduce channel dimension by element-wise addition, as shown in Figure 5 and Figure 6. This whole process is repeated until the finest resolution map is generated. 

As specifically depicted in Figure 5, when we trace the top-down path in the FPN, a 1 × 1 convolutional filter is applied to reduce C5 channel depth to 256-d to create M5, and a subsequent 3 × 3 convolution is performed to create P5, which becomes the first feature map layer used for object prediction [15]. For each subsequent layer, we up-sample the previous layer by 2 using nearest neighbor up-sampling and apply a 1 × 1 convolution to the corresponding feature maps from ResNet. Then, we add the up-sampled feature map to the output feature map by 1 × 1 convolution based on element-wise summation and repeat this process with 3 × 3 convolution to obtain the corresponding feature map layer.

Two subnets named classification subnet and box regression subnet with different tasks are applied to predict results, as shown in Figure 4b. FPN is not an object detector but a feature detector that works with the object detector. Therefore, multiple feature map layers are extracted by the FPN and then fed into the region proposal network (RPN), for example, to detect objects. The RPN then applies 3 × 3 convolutions over the feature maps followed by separate 1 × 1 convolution for class predictions and bounding box regressions. In our study, the classification subnet predicts the probability of object presence at each spatial position for each of the A anchors and K object classes; the parameters of this subnet are then shared between all pyramid levels. The subnet takes an input feature map with C channels from a pyramid level and applies four 3 × 3 convolutional layers with C filters followed by a rectified linear unit (ReLU) activation function. Finally, sigmoid function activations are attached to the output KA binary predictions per spatial location. This subnet implements focal loss (*FL*) [15] as calculated in Equation (4) as the loss function. The focal loss is the reshaping of cross entropy (*CE*) loss in Equation (3) such that it down-weights the loss assigned to well-classified samples; it also focuses training on a sparse set of hard samples and prevents a large number of easy negatives from overwhelming the detector during training [15].

Meanwhile, the box regression subnet is implemented similar to the classification subnet, but the parameters are not shared. The output of this subnet is the object location with respect to anchor box if an object exists, and it terminates in 4A linear outputs per spatial location compared to the KA outputs of the classification subnet with K is number of classes and A is number of anchors. Smooth *L*_1_ loss (Equation (5)) with a sigma of 3 is applied as the loss function to this part of the sub-network [39]:

(3) $$CE\left(p,\;y\right)=\{\begin{array}{l} -log\left(p\right)\;\;\;\;\;\;\;,\;\;if\;y=1\\ -log\left(1-p\right),\;\;\;otherwise \end{array}$$

(4) $$FL\left(p_{t}\right)=\;-1{\left(1-p_{t}\right)}^{\mu }\;log\left(p_{t}\right)$$

(5) $$L_{1;smooth}=\{\begin{array}{l} \left|x\right|\;\;\;\;,\;\;\;if\;\left|x\right|>\alpha \;\;\;\;\;\;\;\;\\ \frac{1}{\left|\alpha \right|}x^{2},\;\;\;else\;if\;\left|x\right|\leq \alpha \end{array}$$

In Equation (3), y∈{±1} defines the ground-truth class, and p∈[0,1] is the model’s estimated probability for the class with label y=1. $p_{t}$ is p if y=1 whereas $p_{t}$ is 1−p if y=−1 [15]. In addition, μ∈[0,5]. While focal loss function adds a modulating factor ${\left(1-p_{t}\right)}^{\mu }$ to the *CE* loss, a tunable focusing parameter μ≥0 and μ value can smoothly adjust the rate at which easy examples are down-weighted to reduce the loss contribution [15]. In Equation (5), α is a hyper-parameter and usually set to 1. The variable x is the *L*_1_ distance between two vectors.

## 5. Experimental Results

### 5.1. Experimental Dataset

**Figure 7:**
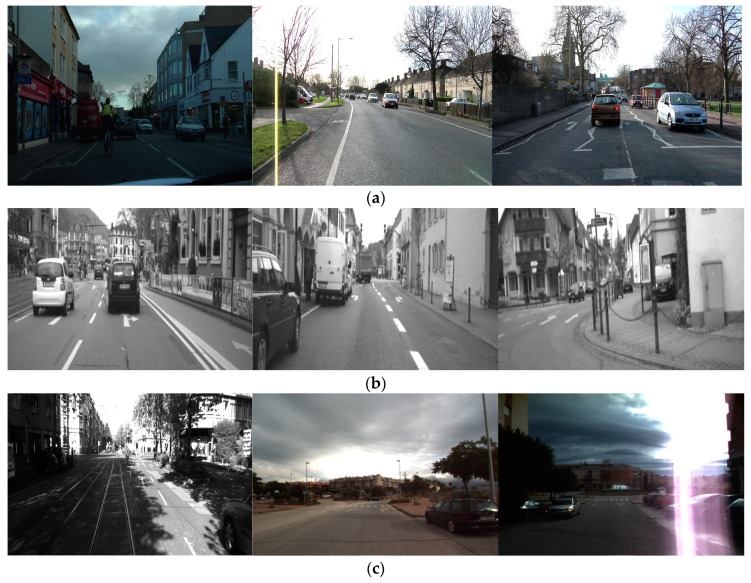
Examples of images from the open datasets. (**a**) Cambridge dataset. (**b**) Daimler dataset. (**c**) Malaga urban dataset.

We trained and tested the network model with various datasets under different illumination and complex conditions. The Cambridge dataset contains four sub-datasets (Seq01TP, Seq06R0, Seq16E5, and Seq05VD) captured in the UK, and the size of each image is 960 × 720 pixels [18]. The Daimler dataset includes the sub-datasets (Test2, Train1, and Train3) with sizes of 1012 × 328 pixels each [17]. In addition, the Malaga urban dataset contains images captured in urban roads in Spain under various illumination conditions, and the size of each image is 800 × 600 pixels [16]. For the experiments, we selected 3572, 898, and 9120 images from the Cambridge, Daimler, and Malaga urban datasets, respectively, by excluding images where eight classes of road markings to be detected and classified in our research (Figure 1) were not included. Example images of each dataset are shown in Figure 7, and Table 3 summarizes the descriptions of each dataset.

We provide the manually annotated information of road markings for the Malaga urban dataset, Daimler dataset, and Cambridge dataset through [25]. In addition, we provide the proposed training models based on different backbones with or without pre-trained weights to other researchers for fair comparison purposes through [25].

### 5.2. Training Process

**Figure 8:**
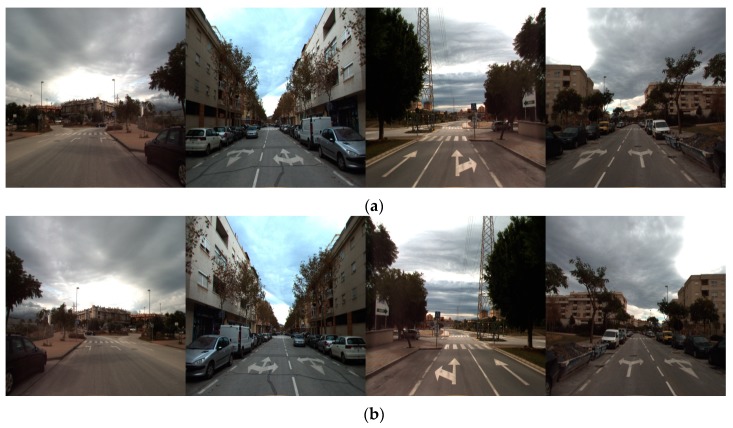
Examples of augmented images. (**a**) Original images. (**b**) Flipped images.

For evaluating the performance of deep RetinaNet-based road marking detection and classification, we performed the experiments based on a two-fold cross validation. The database of all images was divided into two subsets for training and testing, respectively, and the whole process was repeated anew by swapping these subsets. The overall performance was measured based on the average of the obtained results from the two-fold validation scheme. Usually, large datasets are required to train deep CNNs for better performance and to avoid overfitting; thus, data augmentation was used to increase the training data in this work [9]. There should not be large changes in the geometries of the original road markings based on the front-viewing camera after data augmentation. Therefore, data augmentation was performed only by image shifting ±4 pixels and horizontal flipping in our experiments. This kind of data augmentation has been widely used in previous research [9]. Each original training image was horizontally and vertically shifted by (−4, −4), (0, −4), (+4, −4), (−4, 0), (0, 0), (+4, 0), (−4, +4), (0, +4), (+4, +4), thus generating nine versions of the image by simple image shifting. In addition, by horizontally flipping each image, two versions of each shifted image were generated via data augmentation, as shown in Figure 8, for a total of 18 versions per original image; the total number of images thus obtained is summarized in Table 4. Data augmentation was performed only for the training images, and original images were used for testing.

**Figure 9:**
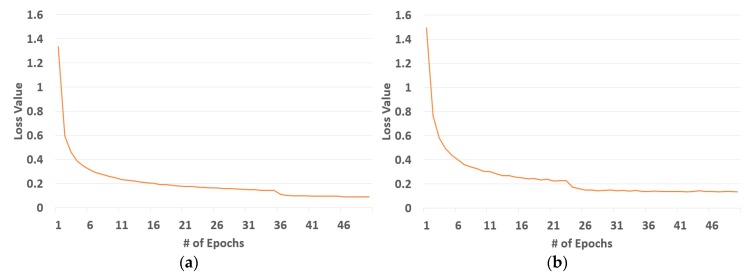
Convergence graphs depicting losses from the training process. (**a**) First set. (**b**) Second set.

For the training of deep RetinaNet, a method for stochastic optimization (Adam) was used, and the training parameters were as follows: the epoch number was 50, number of iterations within each epoch was 10,000, learning rate was initialized at 0.0001 with reduction factor of 0.1, and the two losses were controlled; the regression loss used smooth L1 and the classification loss used *FL*. We performed the training using a desktop computer with Intel Core^TM^ i7 processor of speed 3.47 GHz, 12 GB main memory, and NVIDIA GeForce GTX 1070 graphics card including 1920 compute unified device architecture (CUDA) cores and 8 GB graphics memory [40]. The algorithm was implemented by Keras-Tensorflow [41] on the Ubuntu 16.04 operating system [42]. More specifically, we setup python version 3.5, Tensorflow-GPU version 1.9, NVIDIA CUDA^®^ toolkit 9.0, and NVIDIA CUDA^®^ deep neural network library (cuDNN) version 7.0 on the computer. The training loss converged to 0 for each repetition, as shown in Figure 9, which implies that our network was sufficiently trained with the augmented data.

### 5.3. Testing of the Proposed Method

#### 5.3.1. Accuracies According to Databases and Classes

Testing was performed on both a desktop computer with the same configuration as the training system and a Jetson TX2 embedded system [24]. The testing results are calculated as average values of the two-fold cross validations. To measure the accuracies of road marking detection and classification, the ground-truth positions of the bounding boxes including each road marking were manually annotated in the images and then compared for the overlapping regions between the detected and ground-truth bounding boxes. In our method, we only consider whether the detected road marking is correct or not, so true negative (TN) data are not obtained (i.e., ground-truth data of a non-object); thus, TN errors are 0% in our experiments. Other kind of errors such as true positive (TP), false positive (FP), and false negative (FN) are calculated to obtain precision, recall, accuracy, and F_score, as shown in Equations (6)–(9) [43]. The number of TP, FP, and FN errors are represented as #TP, #FP, and #FN, respectively:

(6) $$\mathrm{Precision}=\frac{\#\mathrm{TP}}{\#\mathrm{TP}+\#\mathrm{FP}}$$

(7) $$\mathrm{Recall}=\frac{\#\mathrm{TP}}{\#\mathrm{TP}+\#\mathrm{FN}}$$

(8) $$\mathrm{Accuracy}=\frac{\#\mathrm{TP}+\#\mathrm{TN}}{\#\mathrm{TP}+\#\mathrm{FP}+\#\mathrm{TN}+\#\mathrm{FN}}$$

(9) $$F\_\mathrm{score}=2\;\times \frac{\mathrm{Precision}\times \mathrm{Recall}}{\mathrm{Precision}+\mathrm{Recall}}$$

Table 5 and Table 6 show the results of detection and classification using our deep RetinaNet with the revised ResNet50 using the initial weights pre-trained by the ImageNet database [10,44]. Table 5 shows the results according to each dataset, and the accuracies for each class are shown in Table 6. 

**Figure 10:**
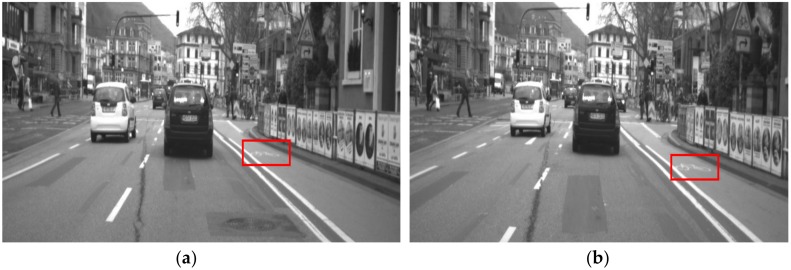
Faded bike markings on the road (red box). (**a**) Example 1 and (**b**) example 2.

The reason why the detected result of Bike marking is low in Table 6 is that these markings in sub-dataset Test2 in the Daimler dataset are faded, as depicted in Figure 10.

**Figure 11:**
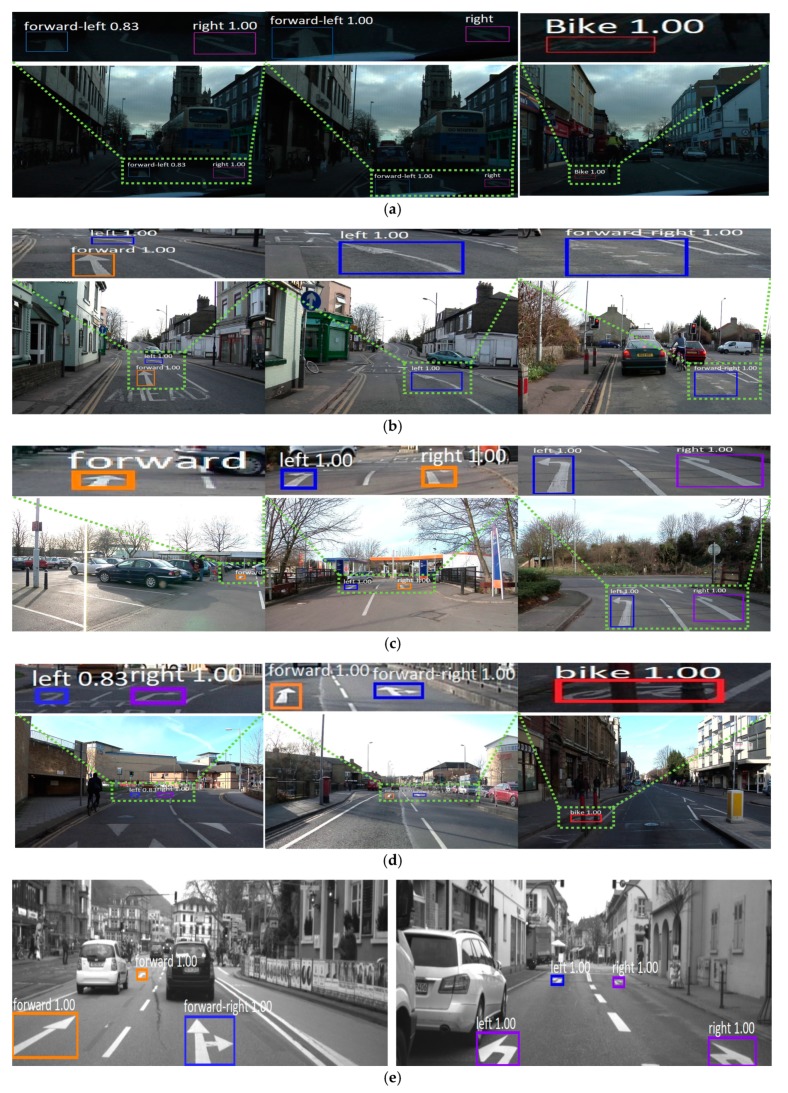

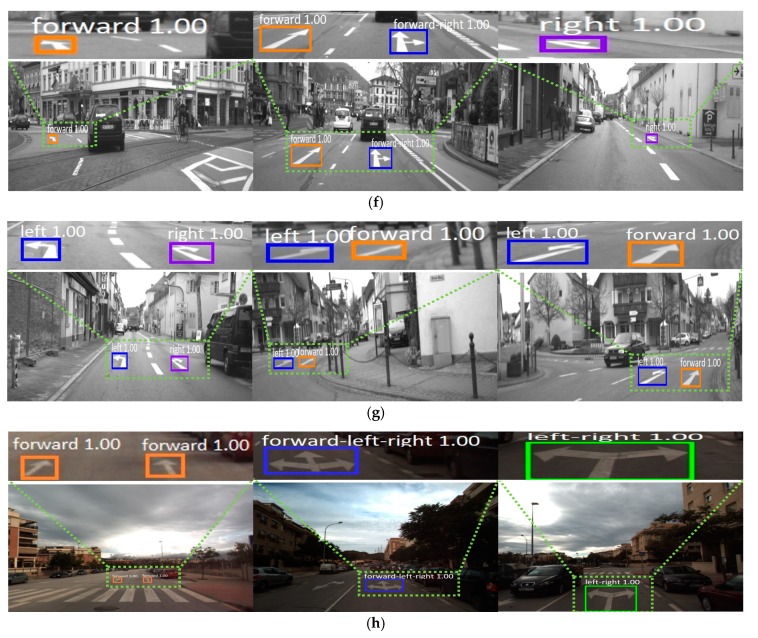
Examples of correct detection and classification cases. (**a**) Seq01TP, (**b**) Seq05VD, (**c**) Seq06R0, and (**d**) Seq16E5 from the Cambridge dataset; (**e**) Test2, (**f**) Train1, and (**g**) Train3 from the Daimler dataset; (**h**) Malaga urban dataset. In (**a**–**h**), true positive cases are shown by the boxes of various colors.

Figure 11 shows the correct detection and classification cases from our deep RetinaNet, which proves that the proposed method can work well under various illumination conditions as well as detect small road markings at a distance. As seen in Figure 11a, road markings can be correctly detected and classified even in low illumination conditions. In Figure 11b,d, our method can also detect road markings even if they are a little faded or occluded. Figure 11c–h shows the cases where multiple road markings are detected correctly. In addition to the label of class category, the detection probability of the object is represented from 0 to 1. For example, “1.00” of Figure 11a shows that the detection probability of the object is 100%.

**Figure 12:**
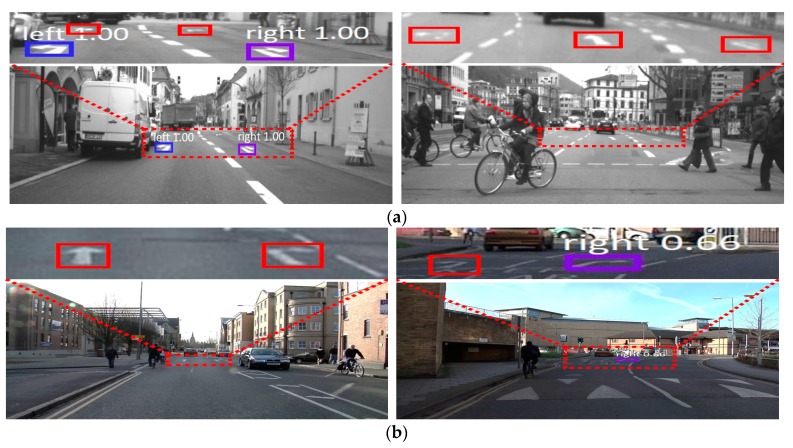
Examples of incorrect detection and classifications. (**a**) Test2 of the Daimler dataset. (**b**) Seq06R0 of the Cambridge dataset. In (**a**,**b**), the red colored boxes with solid lines indicate false negatives whereas the boxes of other colors represent true positives.

Figure 12 shows examples of incorrect detection of road markings (false rejection cases), which are shown as red colored boxes with solid lines. In our research, we train our network with augmented images to avoid overfitting, and there is no case (false acceptance case) in which the road background is incorrectly detected as road marking. However, in some cases where the road objects are small or marking quality is not good, as shown in this figure, the road markings could not be detected. Figure 12 explains why the testing accuracies in these sub-datasets are lower than the others, as summarized in Table 5.

#### 5.3.2. Comparisons of Accuracies by Deep RetinaNet with Those by One-Stage and Two-Stage Methods

As we explained in Section 4.2, deep RetinaNet can work with various backbone CNNs (encoders), such as ResNet (ResNet50 or ResNet101), DenseNet, VGG net-16, and VGG net-19. In this experiment, we compared the deep RetinaNet with ResNet50 with the weights pretrained with ImageNet database (Retina_1) or without the pretrained weights with ImageNet database (Retina_2). In addition, the case where VGG net-16 was used as the encoder (Retina_3) was also compared. Further, other detectors of Faster R-CNN [28,45] as the two-stage method and you only look once version 3 (YOLOv3) [30,46] as the one-stage method were compared. As shown in Table 7, our method for Retina_1 shows higher accuracies than those for Retina_2 and Retina_3 in terms of accuracies and F_score. Furthermore, our method for Retina_1 outperforms the Faster R-CNN [28,45] (two-stage method) and YOLOv3 [30,46] (one-stage method).

**Figure 13:**
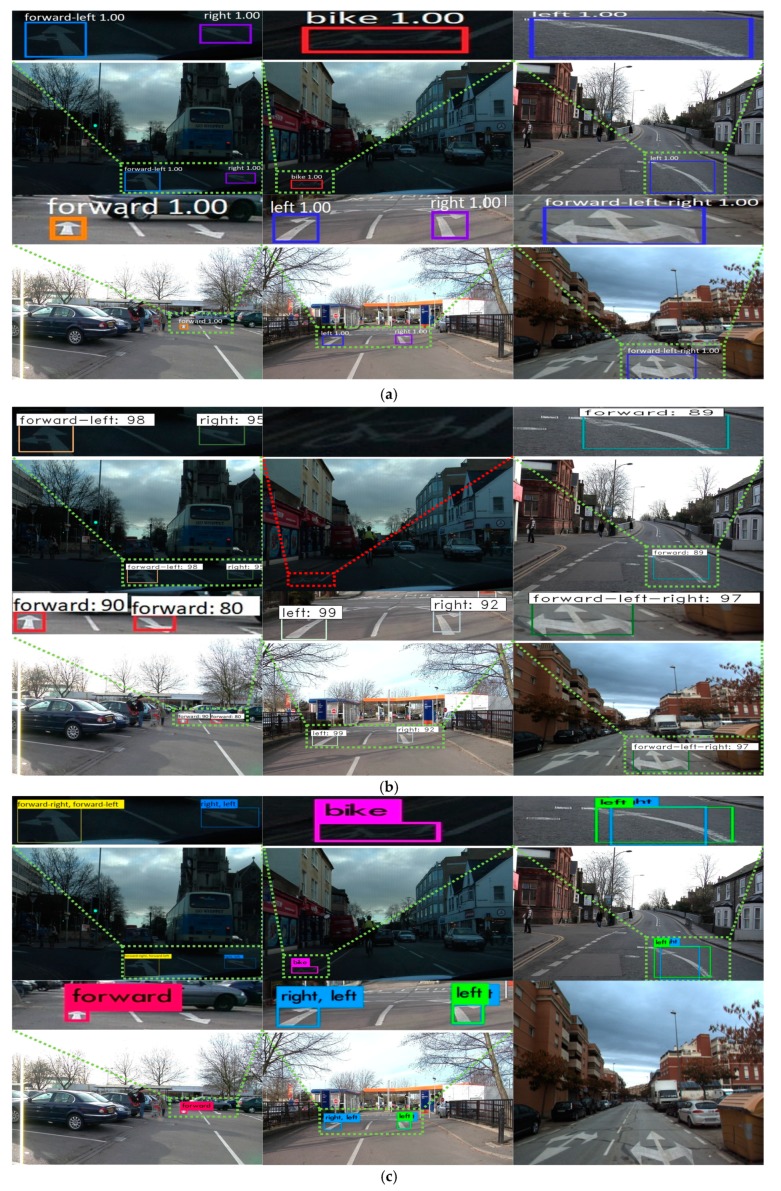
Comparison of road marking detection: (**a**) Proposed method. (**b**) Faster R-CNN. (**c**) YOLOv3.

Figure 13 shows examples of road marking detection by our method, Faster R-CNN, and YOLOv3. As shown in Figure 13, although there are detection errors for Faster R-CNN and YOLOv3, our method can correctly detect and classify them. As shown in the upper-center image of Figure 13b, false rejection case of bike marking occurs by Faster R-CNN. In addition, false positive case for inverted forward arrow occurs in the bottom-left image of Figure 13b. As shown in the upper-left, upper-right, and bottom-center images of Figure 13c, multiple detections happen on road markings as false positive cases by YOLOv3. As shown in the bottom-right image of Figure 13c, false negative cases happen by YOLOv3 as well.

#### 5.3.3. Comparisons of Accuracies Using Original Image with Those by Birds-Eye View Image

**Figure 14:**
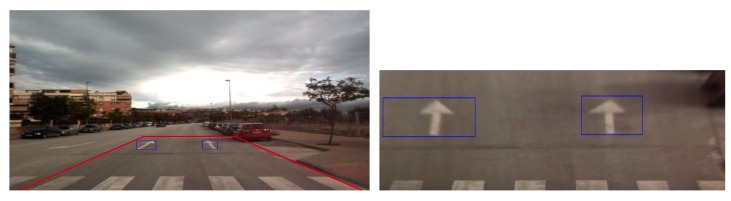
Examples of the original (**left** image) and its corresponding IPM images (**right** image).

**Figure 15:**
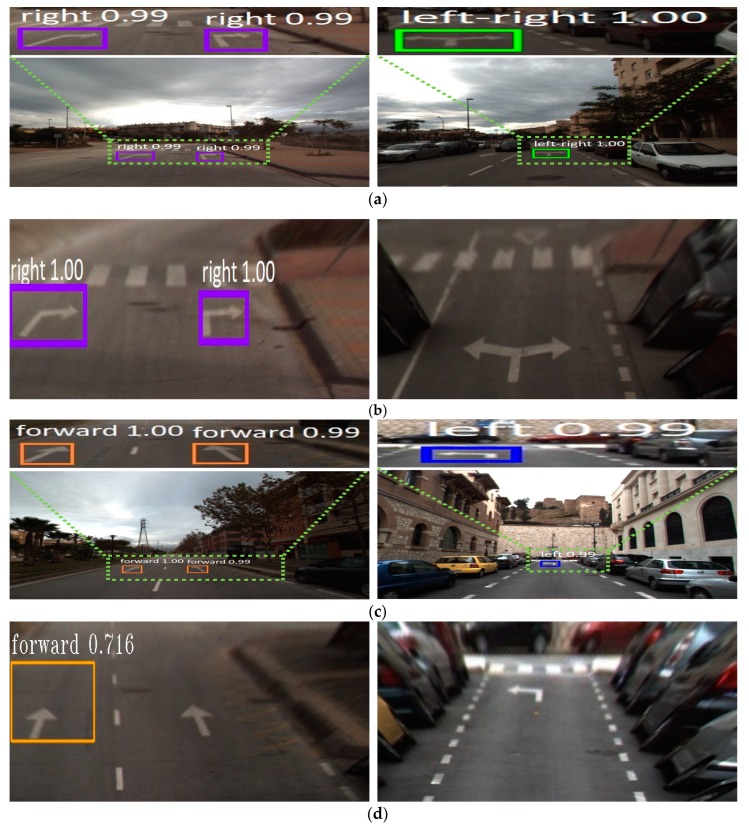
Examples of detection results by the original or IPM images. Result by (**a**) original image, and that by (**b**) IPM image of (**a**). Result by (**c**) original image, and that by (**d**) IPM image of (**c**).

As described in Section 2, existing research have used a bird’s-eye view image obtained by inverse perspective mapping (IPM) pre-processing to detect road markings because such an image can reduce the complexity of the original image by representing the curve as a straight line [47]. The IPM projects the original front-view image obtained from the camera mounted on the vehicle on the bird’s-eye view image so that the local route map (which is typically also in bird’s-eye view) can be fused with the projected image. The IPM projection assumes that the vehicle performs minor pitch or roll movements during the operation (this assumption is valid in most urban driving scenarios involving low-speed, fixed-route vehicles such as buses). Therefore, a fixed, pre-determined projection matrix can be used for the IPM [48]. However, large pitch and roll movements of the vehicle can cause errors if the same fixed projection matrix is used for the IPM, which can result in large deviations of map fusion and detection failure in the subsequent processes [49,50]. In addition, the fixed projection matrix has another disadvantage as the IPM image works well only in small ROIs with road markings at a close distance [47] as shown in Figure 14. A solution to this problem is to install an inertial measurement unit and measure the real-time attitudes of the vehicle, so that these measurements can be used to dynamically compensate for the projection matrix of the IPM. Considering this issue, our method uses the original image as the input for the deep RetinaNet without the IPM pre-processing as shown in Figure 3. In the next experiment, we compared the accuracies using the original and IPM images for training and testing our RetinaNet. Based on the results in Table 7, we used RetinaNet with ResNet50 with the weights pre-trained with ImageNet databases (Retina_1) for the experiments. As listed in Table 7, the detection and classification accuracies obtained using the original image are higher than those obtained using the IPM image. Figure 15 shows examples of detection results obtained using the original and IPM images. As shown in the images on the right of Figure 15b, and on the left and right of Figure 15d, false rejection cases occurred in the IPM images even if they were correctly detected in the original images. In the images on the left of Figure 15b,d, some road markings are correctly detected in the IPM images, but their detected boxes are larger than those in the original images, which can cause confusion that both road markings and background are included in the detected box. Based on the experimental results in Table 8, we can find that the IPM projection is difficult to be used for multi-datasets with different parameters of camera installation and the large pitch or roll movements of vehicle during the operation. In addition, we can observe that the IPM method uses fixed and pre-determined projection matrix, and it can be used for small ROIs with road markings at a close distance. These can be obstacle for being adopted to real car application.

#### 5.3.4. Measuring Processing Speed and Evaluation of Embedded Systems

In the next experiment, we compared the processing time of our method with that of Faster R-CNN [28,45] and YOLOv3 [30,46] on a desktop computer. The specifications of the desktop computer are explained in Section 5.2. As described in Table 9, our method is faster than the one and two-stage methods, YOLOv3 and Faster R-CNN, respectively.

**Figure 16:**
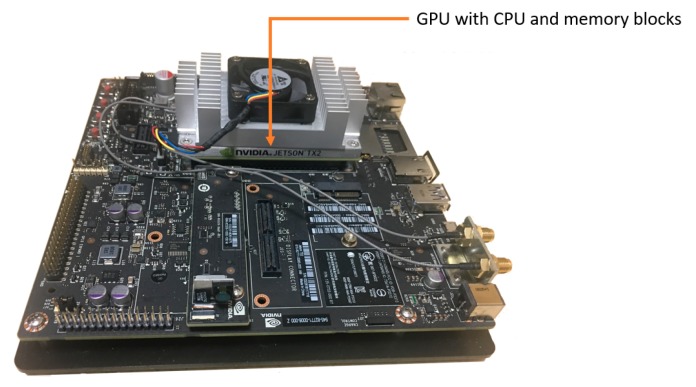
Jetson TX2 embedded system.

In the next experiment, we compared the processing speed of the embedded systems. Considering the application of our method to embedded systems in actual vehicles, we used the Jetson TX2 embedded system [24] as shown in Figure 16 with NVIDIA Pascal^TM^ -family GPU, having 8GB of memory shared between the central processing unit (CPU) and GPU, and 59.7 GB/s of memory bandwidth; it uses less than 7.5 watts of power. The details of the specifications of this board are explained in Table 10. This board has been widely used in an actual car environment for autonomous vehicles. As indicated in Table 11, our method is faster than YOLOv3 and Faster R-CNN on Jetson TX2 embedded systems. The reason why Faster R-CNN has a lower processing speed than our method is that it requires approximately 15.3 billion floating point operations per second (FLOPs) for VGGNet-16, but only 3.8 billion FLOPs are required while using ResNet50 [34] in our deep RetinaNet.

## 6. Conclusions

In this research, we propose a novel one-stage method based on deep RetinaNet that can detect and classify road markings in various conditions and at long-range distances with high accuracy. Testing results obtained from three open databases show that our network model has advantages in terms of accuracy and processing speed when compared with other one and two-stage methods. Our method has also the benefit of high processing time in both the desktop computer environment and embedded system of Jetson TX2 board [24]. Because of using Keras-Tensorflow instead of Matlab (toolbox), our algorithm could be easily ported on an NVIDIA Jetson TX2 embedded system. In addition, the processing speed of our algorithm is fast enough for being operated on both desktop computer and NVIDIA Jetson TX2 embedded system as shown in Table 9 and Table 11 by using Keras-Tensorflow instead of Matlab (toolbox). Through experiments conducted using RetinaNet with various encoders and input image types, we prove the effectiveness of our method. The detection and classification accuracies degrade in some cases with faded bike markings.

As shown in Table 7, the overall precision by our method is higher than that by YOLOv3 whereas the overall recall by YOLOv3 is higher than that by our method. Because the precision usually has the trade-off relationship with recall, the accuracy and F_score considering both precision and recall at the same time have been widely used as the evaluation metrics. As shown in the experiments of Table 7 with eight sub-datasets from three open databases, the accuracy by our method is higher than YOLOv3 with 6 sub-datasets, and the average accuracy by our method with whole sub-datasets is higher than that by YOLOv3. In addition, as shown in the experiments of Table 7, the F_score by our method is higher than YOLOv3 with 6 sub-datasets, and the average F_score by our method with whole sub-datasets is higher than that by YOLOv3. Even with one (Seq06R0) of the remained 2 sub-datasets, our method using VGG net-16 as encoder (Retina_3) shows the same F_score as that by YOLOv3. However, the processing speed by our method is faster than that by YOLOv3 on both desktop computer and embedded system as shown in Table 9 and Table 11. Considering the real-time operation in embedded system of car environment, the low processing time is very important, and we can conclude that our method has more benefit than YOLOv3.

In our future work, we intend to combine our method with image restoration to handle this problem. Instead of processing the whole image, we would segment only road drivable region, perform the geometry transform of the segmented region based on adaptive parameters, and use this transformed region to the input of CNN model for object detection. Combining with image restoration methods, we can reduce the computational complexity by not considering the noises from non-drivable background. Alternatively, we can also consider the method using two different CNN models for detection and classification, respectively. The first CNN model would focus on the detection of object and background. Then, the detail classes of the object would be classified by the second CNN model. By this approach, we can expect that the processing complexity of the proposed RetinaNet which includes the functionalities of detection and classifications of multiple classes can be reduced, which can enhance the overall performance of road marking detection. In addition, we would study the method of making our network lighter so as to operate it at a faster speed on embedded systems.

## Figures and Tables

**Table 1:** Comparisons between existing method and the proposed method for detection and classification of road markings.

Category	Method	Advantage	Disadvantage
Handcrafted features-based	Uses color space different from RGB [7,8] Local adaptive threshold and edge detector [6] Line segment detector [2,3] Uses marking pixel extraction and pattern comparison [5]	No extensive training is required Algorithm is simple with low processing time	Performs well only for specific conditions Intensive pre- and post-processing is required Poor performance under extreme conditions Original image must be converted to bird’s eye view to detect straight edge line segment
Deep features-based	VPGNet [12] used for vanishing point detection and detection and classification of road markings BING and PCANet [19] DVCNN [20] Deep CNN [9,11,21], CNN with RNN [10], and GLAD [22] Uses density-based grouping and shallow CNN [23]	Outperforms handcrafted features-based methods Work well with various shapes and types of road markings in extreme weather conditions	Additional pre-processing is required [10,11,19,20,23] Evaluations were not performed on multiple datasets collected from different countries, including various shapes and types of road markings [9,10,12,19,20,21,22,23]
	Uses one-stage deep CNN (proposed method)	Does not require pre- and post-processing Performs evaluation on multiple datasets collected from different countries, including various shapes and types of road markings	Requires intensive training for deeper CNN model than previous deep features-based methods

**Table 2:** Revised ResNet50 architecture used in our RetinaNet. Each layer is followed by batch normalization (BN) and rectified linear unit (ReLU) activation function. 2/1* means 2 at the first iteration and 1 from the second iteration. The shortcut for a 1 × 1 convolutional filter is included in each layer of Conv2_x, …, Conv5_x. Conv1 is performed with the feature map including a padding of 3, whereas the convolutional filtering of 3 × 3 × depth in Conv2_x, …, Conv5_x is performed with the feature maps including paddings of 1. In all the other cases, the padding number is 0.

Layer Name	#of Iterations	Kernel Size	#of Filters	Stride	Size of Feature Map (Height × Width × Channel)
Input Layer					720 × 960 × 3
Conv1	1	7 × 7 × 3	64	2	360 × 480 × 64
Max pool	1	3 × 3	1	2	180 × 240 × 64
Conv2_x	x3	1 × 1 × 64	64	1	180 × 240 × 64
		3 × 3 × 64	64	1	180 × 240 × 64
		1 × 1 × 64	256	1	180 × 240 × 256
Conv3_x	x4	1 × 1 × 256	128	2/1*	90 × 120 × 128
		3 × 3 × 128	128	1	90 × 120 × 128
		1 × 1 × 128	512	1	90 × 120 × 512
Conv4_x	x6	1 × 1 × 512	256	2/1*	45 × 60 × 256
		3 × 3 × 256	256	1	45 × 60 × 256
		1 × 1 × 256	1024	1	45 × 60 × 1024
Conv5_x	x3	1 × 1 × 1024	512	2/1*	23 × 30 × 512
		3 × 3 × 512	512	1	23 × 30 × 512
		1 × 1 × 512	2048	1	23 × 30 × 2048

**Table 3:** Descriptions of each dataset.

Dataset	Sub Dataset	Image Size (Pixels)	Number of Images (Frames)	Total (Frames)
Cambridge	Seq01TP	960 × 720	216	3572
	Seq05VD		162	
	Seq06R0		1518	
	Seq16E5		1676	
Daimler	Test2	1012 × 328	470	898
	Train1		362	
	Train3		66	
Malaga urban		800 × 600	9120	9120

**Table 4:** Number of images in original training, augmented training, and testing sets (unit: frames).

Dataset	Original Training Set	Augmented Training Set	Testing Set
Cambridge	1786	32,148	1786
Daimler	449	8082	449
Malaga urban	4560	82,080	4560

**Table 5:** Accuracies by our proposed method for different datasets.

Dataset	Sub-Dataset	Precision	Recall	Accuracy	F_score
Cambridge	Seq01TP	1.000	1.000	1.000	1.000
	Seq05VD	0.988	0.904	0.895	0.944
	Seq06R0	0.999	0.864	0.863	0.926
	Seq16E5	0.999	0.953	0.952	0.976
Daimler	Test2	0.989	0.750	0.744	0.853
	Train1	1.000	0.955	0.955	0.977
	Train3	1.000	1.000	1.000	1.000
Malaga		0.993	0.973	0.966	0.983
Average		0.996	0.925	0.922	0.957

**Table 6:** Accuracies by our proposed method for different classes.

Classes		Precision	Recall	Accuracy	F_score
Bike (B)		0.999	0.753	0.752	0.859
Arrow	Forward (F)	0.995	0.873	0.869	0.930
	Forward–left (FL)	0.993	0.989	0.982	0.991
	Forward–left–right (FLR)	0.987	1.000	0.987	0.993
	Forward–right (FR)	0.997	0.945	0.942	0.970
	Left (L)	1.000	0.982	0.982	0.991
	Left–right (LR)	0.967	1.000	0.967	0.983
	Right (R)	0.992	0.948	0.940	0.970
Average		0.991	0.936	0.928	0.961

**Table 7:** Comparisons between proposed method and other methods of different encoders, one-stage and two-stage methods.

Criterion	Methods	Cambridge				Daimler			Malaga	Avg.
		Seq 01TP	Seq 05VD	Seq 06R0	Seq 16E5	Test2	Train1	Train3		
Precision	Ours (Retina_1)	1.000	0.988	0.999	0.999	0.989	1.000	1.000	0.993	0.996
	Ours (Retina_2)	0.985	0.988	0.995	0.997	0.996	1.000	1.000	0.993	0.994
	Ours (Retina_3)	0.992	0.988	1.000	0.997	0.996	1.000	1.000	0.993	0.996
	Faster R-CNN [28,45]	0.966	0.988	0.985	0.974	0.966	0.931	0.955	0.979	0.968
	YOLOv3 [30,46]	0.682	0.628	0.869	0.623	0.841	0.543	0.719	0.771	0.710
Recall	Ours (Retina_1)	1.000	0.904	0.864	0.953	0.750	0.955	1.000	0.973	0.925
	Ours (Retina_2)	0.992	0.94	0.862	0.953	0.739	0.972	1.000	0.973	0.929
	Ours (Retina_3)	0.985	0.904	0.867	0.953	0.744	0.955	1.000	0.973	0.923
	Faster R-CNN [28,45]	0.851	0.883	0.782	0.810	0.859	0.874	1.000	0.498	0.820
	YOLOv3 [30,46]	1.000	0.989	0.999	0.997	0.894	0.919	1.000	0.985	0.973
Accuracy	Ours (Retina_1)	1.000	0.895	0.863	0.952	0.744	0.955	1.000	0.966	0.922
	Ours (Retina_2)	0.977	0.884	0.859	0.950	0.737	0.972	1.000	0.966	0.918
	Ours (Retina_3)	0.978	0.895	0.867	0.951	0.742	0.955	1.000	0.966	0.919
	Faster R-CNN [28,45]	0.826	0.874	0.771	0.793	0.834	0.821	0.955	0.493	0.796
	YOLOv3 [30,46]	0.682	0.624	0.868	0.622	0.765	0.518	0.719	0.763	0.695
F_score	Ours (Retina_1)	1.000	0.944	0.926	0.976	0.853	0.977	1.000	0.983	0.958
	Ours (Retina_2)	0.989	0.963	0.924	0.975	0.848	0.986	1.000	0.983	0.958
	Ours (Retina_3)	0.989	0.944	0.929	0.975	0.852	0.977	1.000	0.983	0.956
	Faster R-CNN [28,45]	0.905	0.932	0.870	0.884	0.909	0.901	0.977	0.661	0.880
	YOLOv3 [30,46]	0.811	0.769	0.929	0.767	0.867	0.683	0.837	0.865	0.816

**Table 8:** Comparative experimental results using the original and IPM images.

Criterion	Methods	Cambridge				Daimler			Malaga	Avg.
		Seq 01TP	Seq 05VD	Seq 06R0	Seq 16E5	Test2	Train1	Train3		
Precision	Original image	1.000	0.988	0.999	0.999	0.989	1.000	1.000	0.993	0.996
	IPM image [48]	0.957	0.959	0.994	0.991	0.973	0.964	0.927	0.990	0.969
Recall	Original image	1.000	0.904	0.864	0.953	0.750	0.955	1.000	0.973	0.925
	IPM image [48]	0.827	0.823	0.848	0.847	0.712	0.802	0.821	0.816	0.812
Accuracy	Original image	1.000	0.895	0.863	0.952	0.744	0.955	1.000	0.966	0.922
	IPM image [48]	0.797	0.795	0.844	0.840	0.698	0.779	0.771	0.809	0.792
F_score	Original image	1.000	0.944	0.926	0.976	0.853	0.977	1.000	0.983	0.958
	IPM image [48]	0.887	0.886	0.916	0.913	0.822	0.876	0.871	0.895	0.883

**Table 9:** Processing time per each frame in the desktop computer environment (unit: millisecond).

Dataset	Sub Dataset	Processing Time		
		Proposed Method	Faster R-CNN [28,45]	YOLOv3 [30,46]
Cambridge	Seq01TP	50	291	49
	Seq05VD	50	297	50
	Seq06R0	47	279	47
	Seq16E5	50	319	50
Daimler	Test2	31	656	40
	Train1	35	631	40
	Train3	37	668	40
Malaga		42	278	39
Average		42.75	427.38	44.38

**Table 10:** Specifications of the Jetson TX2 embedded system.

Jetson TX2 Embedded System	
GPU	NVIDIA Pascal^TM^, 256 CUDA cores
CPU	HMP Dual Denver 2 (2 MB L2) + Quad ARM^®^ A57 (2 MB)
Memory	8 GB
Data storage	32 GB
Operating system	Linux for Tegra R28.1 (L4T 28.1)
Dimensions (width × height × depth)	50 mm × 87 mm × 10.4 mm

**Table 11:** Processing time per each frame on Jetson TX2 embedded system (unit: millisecond).

Dataset	Sub Dataset	Processing Time		
		Proposed Method	Faster R-CNN [24,37]	YOLOv3 [27,38]
Cambridge	Seq01TP	50	297	50
	Seq05VD	57	297	54
	Seq06R0	54	286	50
	Seq16E5	54	319	52
Daimler	Test2	38	662	42
	Train1	39	638	45
	Train3	38	675	43
Malaga		44	281	43
Average		46.75	431.875	47.375
